# MUCIN 1 confers inflammatory memory of tyrosine kinase inhibitor resistance in non-small cell lung cancer

**DOI:** 10.1038/s41392-025-02482-7

**Published:** 2025-11-28

**Authors:** Shinkichi Takamori, Naoki Haratake, Atrayee Bhattacharya, Chie Kikutake, Hiroki Ozawa, Keisuke Shigeta, Ayako Nakashoji, Hideko Isozaki, Mototsugu Shimokawa, Mikita Suyama, Asato Hashinokuchi, Kazuki Takada, Gouji Toyokawa, Yuichi Yamada, Tomoyoshi Takenaka, Kenichi Taguchi, Masafumi Yamaguchi, Tomoharu Yoshizumi, Aaron N. Hata, Donald Kufe

**Affiliations:** 1 Department of Medical Oncology, Dana-Farber Cancer Institute, Harvard Medical School, Boston, MA USA; 2 Department of Thoracic and Breast Surgery, Oita University Faculty of Medicine, Yufu, Oita, Japan; 3 Division of Bioinformatics, Medical Institute of Bioregulation, Kyushu University, Fukuoka, Japan; 4 Department of Medicine, Massachusetts General Hospital, Harvard Medical School, Boston, MA USA; 5 Department of Biostatistics, Graduate School of Medicine, Yamaguchi University, Yamaguchi, Japan; 6 Department of Thoracic Oncology, NHO Kyushu Cancer Center, Fukuoka, Japan; 7 Department of Surgery and Science, Graduate School of Medical Sciences, Kyushu University, Fukuoka, Japan; 8 Fukuoka Pathology Clinic, Fukuoka, Japan; 9 Cancer Pathology Laboratory, NHO Kyushu Cancer Center, Fukuoka, Japan

**Keywords:** Cancer therapy, Lung cancer

## Abstract

Resistance of NSCLCs to osimertinib, an EGFR tyrosine kinase inhibitor (TKI), is mediated by pleotropic mechanisms that pose a significant challenge for subsequent treatment. We report that the oncogenic MUC1-C/M1C protein confers resistance to osimertinib by regulating the STAT1 and interferon (IFN) type I/II pathways. Studies of osimertinib-resistant NSCLC cell lines selected for growth in the absence of drug demonstrate dependence on MUC1-C and the STAT1 pathway for memory of the refractory phenotype. This inflammatory memory of TKI resistance is mediated through activation of the *MUC1* gene at (i) a proximal enhancer-like signature 1 (pELS-1) by MUC1-C and STAT1 and (ii) a pELS-2 by MUC1-C, JUN/AP-1, and PBAF. Our results further reveal that the MUC1-C-driven STAT1 inflammatory response promotes resistance of patient-derived (i) EGFR mutant NSCLC cells with *MET* amplification to the combination of osimertinib+MET TKIs, and (ii) EGFR(T790M/C797S) NSCLC cells to the 4th generation EGFR TKI TQB3804. Of clinical significance, we report that NSCLC cells dependent on MUC1-C for TKI resistance are druggable with an antibody-drug conjugate (M1C ADC) in vitro and in a PDX tumor model. These findings demonstrate that MUC1-C (i) is essential for TKI resistance of NSCLC cells by driving an inflammatory memory response and (ii) is a target for M1C ADC treatment of TKI-refractory NSCLCs.

## Introduction

Osimertinib is an epidermal growth factor (EGFR) tyrosine kinase inhibitor (TKI) that targets EGFR with TKI-sensitizing and T790M resistance mutations.^1^ Osimertinib has exhibited superiority as compared to standard EGFR-TKIs in untreated patients with *EGFR*-mutated advanced NSCLCs.^2,3^ Osimertinib is also effective in prolonging survival in patients with surgically resected *EGFR*-mutant NSCLCs.^4–7^ The AURA and FLAURA trials established the superiority of osimertinib as the initial choice for the treatment of NSCLCs harboring EGFR mutations.^8^ These advances notwithstanding, NSCLCs treated with osimertinib consistently develop acquired resistance, often within 10-20 months.^8^ Acquired resistance to osimertinib has been attributable to on- and off-target mechanisms that include (i) *MET* gene amplification, (ii) EGFR C797S and other second-site mutations, (iii) the epithelial-mesenchymal transition (EMT), and (iv) activation of the SHP2, AKT, and ERK pathways.^9–14^ Osimertinib is being combined with MET inhibitors capmatinib and savolitinib to circumvent resistance associated with *MET* amplification and other MET-based mechanisms.^8^ Fourth generation EGFR inhibitors, such as EAI045 and TQB3804, are also under development to overcome EGFR C797S-mediated resistance.^9^ Noteworthy is that no identifiable mutations have been identified in ~50% of NSCLCs with acquired osimertinib resistance.^11,15^ The findings that mechanisms of acquired resistance to osimertinib are highly pleotropic have posed a significant challenge for identifying subsequent treatment options to circumvent the poor clinical outcomes in this setting. Antibody-drug conjugates (ADCs) targeting EGFR, MET, and TROP2 represent another strategy for the treatment of osimertinib-resistant EGFR-mutant NSCLCs; however, there are no approved ADCs for this patient population.

The *MUC1* gene appeared in mammals to protect barrier tissues, such as respiratory tract epithelia, that interface with the external environment and are subject to biotic and abiotic insults.^16–18^ *MUC1* encodes a non-mucin transmembrane MUC1-C (M1C) protein that is activated by inflammation and drives cell lineage plasticity necessary for wound healing.^16–18^ MUC1-C thus responds to loss of homeostasis by suppressing polarity and by inducing inflammatory, proliferative, and repair responses.^16–18^ In conferring the inflammatory response, MUC1-C binds to STAT1 and activates the inflammatory interferon (IFN) type I and II pathways.^19–22^ MUC1-C thereby drives the induction of downstream IFN-stimulated genes (ISGs) that encode effectors of anti-viral defenses, anti-proliferative pathways, and adaptive immunity.^23^ In apposition to these protective functions, prolonged activation of MUC1-C by chronic inflammation in barrier epithelia can drive cancer progression.^17,18^ Along these lines, MUC1-C-induced chronic activation of ISGs, such as MX1 and OAS1, confers DNA damage resistance and immune evasion.^20–22^ ISGs also include the *APOBEC3* genes that evolved in mammals to counteract retroviral replication and have been linked to mutagenesis and treatment resistance in cancer.^24^

The significance of MUC1-C in cancer development is supported by a role in integrating inflammatory signaling with epigenetic reprogramming.^18^ MUC1-C regulates Polycomb Repressive Complex 1 (PRC1) and 2 (PRC2).^25–27^ MUC1-C also activates the BAF and PBAF chromatin remodeling complexes,^28–30^, which oppose PRC1/2,^31,32^ and contribute to global changes in differentially accessible regions (DARs) throughout the genomes of cancer cells.^29^ MUC1-C-induced DARs align with differentially expressed genes (DEGs) regulated by the JUN/AP-1 family of TFs.^29^ MUC1-C thereby integrates chromatin remodeling in cancer cells with activation of JUN/AP-1 TFs in driving expression of stemness-associated genes.^29,33^ MUC1-C also activates the COMPASS family of H3K4 methyltransferases that counteract PRC1/2 functions.^31,32,34^ Epigenetic reprogramming is required for the regulation of homeostasis, stem cell plasticity, and the wound healing response.^35^ Epigenetic reprogramming also contributes to memory, which enables barrier tissues to remember insults and more effectively inform responses to subsequent exposures.^36^ Inflammatory memory has been studied in cancer models of responsiveness to immunotherapy;^37,38^, whereas less is known about the involvement of memory in the response to targeted therapies.

To address this unexplored issue, we leveraged the recent findings that MUC1-C acts as a common effector of acquired osimertinib resistance in NSCLC.^39^ These studies demonstrated that MUC1-C is upregulated in osimertinib drug-tolerant persister cells and drives activation of p-EGFR, p-ERK, and p-AKT signaling.^39^ Remarkably, MUC1-C was found to be required for osimertinib resistance in NSCLC cells associated with (i) the EMT phenotype, (ii) MET amplification, and (iii) the secondary EGFR(T790M/C797S) mutation.^39^ Moreover, targeting MUC1-C genetically and pharmacologically in these multiple models reversed osimertinib resistance.^39^ How MUC1-C drives pleotropic mechanisms of osimertinib resistance was not clear. The present work has addressed this conundrum by demonstrating that MUC1-C functions as a master regulator of a NSCLC cell memory response to osimertinib resistance. We further demonstrate that the MUC1-C-driven memory response is conferred by activation of the STAT1 pathway and previously unrecognized *MUC1* inflammatory memory domains. The novelty of these findings resides in having identified that MUC1-C is (i) an effector of a NSCLC STAT1-mediated inflammatory memory pathway that drives resistance to EGFR-TKIs and (ii) a target with an M1C ADC for the treatment of TKI-refractory NSCLC tumors.

## Results

### MUC1-C regulates the IFN type I and II pathways in establishing TKI resistance

**Fig. 1:**
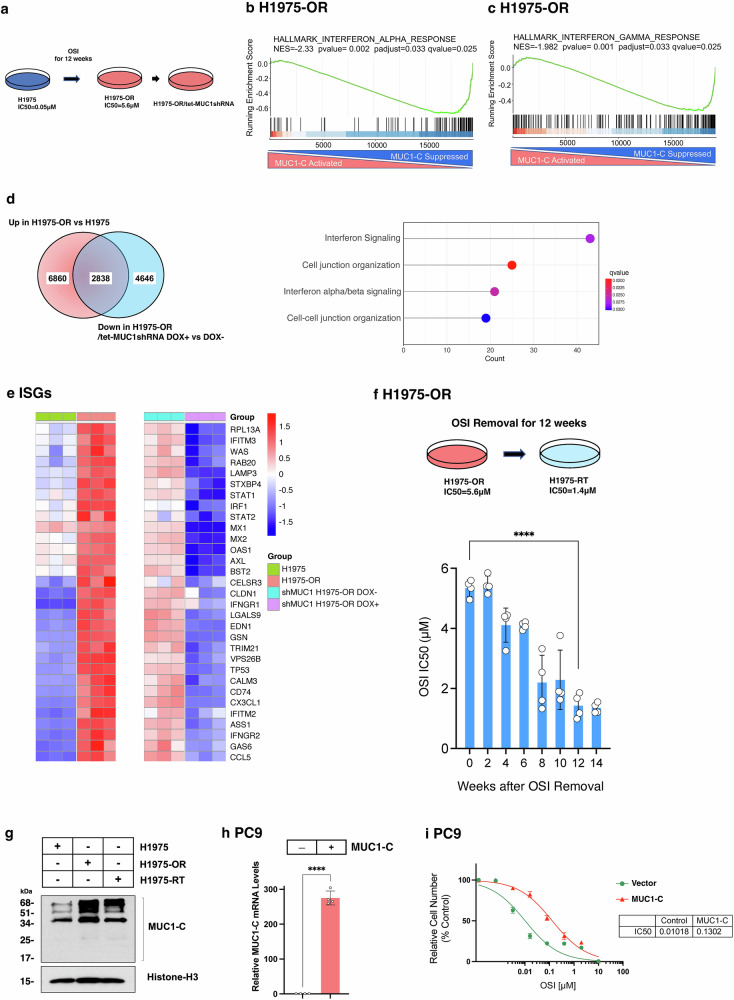
MUC1-C-dependent upregulation of inflammatory pathways in osimertinib-resistant H1975-OR vs parental H1975 cells. **a** H1975 cells were treated with increasing osimertinib concentrations for 12 weeks to select for osimertinib-resistant H1975-OR cells. **b**, **c** RNA-seq was performed on biological triplicates of H1975-OR/tet-MUC1shRNA cells treated with vehicle or DOX for 7 days. GSEA of the datasets using the indicated HALLMARK gene signatures. **d** Venn diagram of 2838 shared genes upregulated in H1975-OR vs H1975 cells and downregulated in H1975-OR cells with MUC1-C silencing. Analysis of the 2838 shared genes identifies interferon signaling and cell junction organization pathways. **e** Heatmap of selected genes upregulated in H1975-OR vs H1975 cells and downregulated by silencing MUC1-C in H1975-OR cells. **f** H1975-OR cells grown in the absence of osimertinib for the indicated weeks were analyzed for osimertinib sensitivity by Alamar Blue staining (Supplementary Fig. 1e). The results (mean±SD of 4 determinations) are expressed as the osimertinib IC50 μM values. **g** Chromatin from H1975, H1975-OR, and H1975-RT cells was immunoblotted with antibodies against the indicated proteins. **h**. PC9 cells transfected with an empty control vector (PC9/vector) or one expressing MUC1-C (PC9/MUC1-C) were analyzed for MUC1-C mRNA levels by qRT-PCR. The results (mean±SD of 4 determinations) are expressed as relative levels compared to that obtained for untreated cells (assigned a value of 1). **i** PC9/vector and PC9/MUC1-C cells were treated with the indicated concentrations of osimertinib for 3 days and analyzed for cell viability by Alamar Blue staining. The results (mean±SD of 4 determinations) are expressed as relative cell number (% control) compared to that for PC9/vector cells

Resistance of NSCLC cells to osimertinib is associated with pleotropic mechanisms that include induction of EMT, *MET* amplification, and the EGFR C797S mutation. Previous work discovered that MUC1-C is a common driver of osimertinib resistance; however, the mechanistic basis for this finding was not addressed in those studies.^39^ To explore this issue, we studied H1975 NSCLC cells exposed to increasing concentrations of osimertinib over 12 weeks to establish a stable osimertinib-resistant (H1975-OR) cell phenotype (Fig. 1a; OSI IC50 = 5.6 μM).^39^ Comparison of transcriptomes from H1975-OR vs H1975 cells identified upregulation of the HALLMARK INFLAMMATORY RESPONSE gene signature (Supplementary Fig. 1a and 1b). We also found that silencing MUC1-C in H1975-OR cells significantly suppresses the HALLMARK INTERFERON ALPHA RESPONSE and HALLMARK INTERFERON GAMMA RESPONSE gene signatures (Fig. 1b and c; Supplementary Fig. 1c and 1 d), indicating that MUC1-C regulates the inflammatory IFN type I and II pathways in association with establishing osimertinib resistance. Of the 6860 genes upregulated in H1975-OR vs H1975 cells and the 4646 genes downregulated in MUC1-C-silenced H1975-OR cells, we identified 2838 common genes (Fig. 1d). Pathway analysis of this shared 2838 geneset uncovered activation of (i) IFN alpha/beta signaling, and (ii) cell junction organization (Fig. 1d). Further analysis of the shared MUC1-C-dependent genes identified STAT1, STAT2, IRF1 and multiple IFN stimulated genes (ISGs) that contribute to (i) the IFN-related DNA damage resistance gene signature (IRDS),^40^ and (ii) inflammatory memory and immune evasion (Fig. 1e).^41^

To extend these studies, H1975-OR cells were grown in the absence of osimertinib to select for a drug-sensitive phenotype (Fig. 1f). Under these conditions, H1975-RT cells were recovered from H1975-OR cells after 12 weeks of drug holiday with an osimertinib IC50 = 1.4 μM that was stably maintained over continued passage (Fig. 1f; Supplementary Fig. 1e). Silencing MUC1-C in H1975-RT cells with different MUC1sgRNAs downregulated (i) MUC1-C and STAT1 expression (Supplementary Fig. 1f) and (ii) decreased the osimertinib IC50 to 0.05 μM (Supplementary Fig. 1g), which is equivalent to that in parental H1975 cells, indicating that MUC1-C is necessary for residual osimertinib resistance in the H1975-RT phenotype. MUC1-C localizes to chromatin predominantly as 34 kDa homodimers and higher order structures, where they interact with TFs and effectors of epigenetic reprogramming.^39,42,43^ Of interest in this regard, chromatin MUC1-C levels were increased in H1975-OR vs H1975 cells and suppressed in H1975-RT vs H1975-OR cells (Fig. 1g), indicating that localization of MUC1-C to chromatin associates with osimertinib resistance. As a control, treatment of the H1975 cell chromatin fraction with dithiothreitol (DTT) reduced MUC1-C multimers to the 17 kDa monomer (Supplementary Fig. 1h), consistent with the formation of MUC1-C multimers by disulfide bonds.^44^ For corroboration with a gain-of-function model, we overexpressed MUC1-C in PC9 NSCLC cells (Fig. 1h) and found that upregulation of MUC1-C levels decreases sensitivity to osimertinib treatment (Fig. 1i), confirming that MUC1-C confers the TKI resistant phenotype.

### MUC1-C is necessary for memory of TKI resistance

**Fig. 2:**
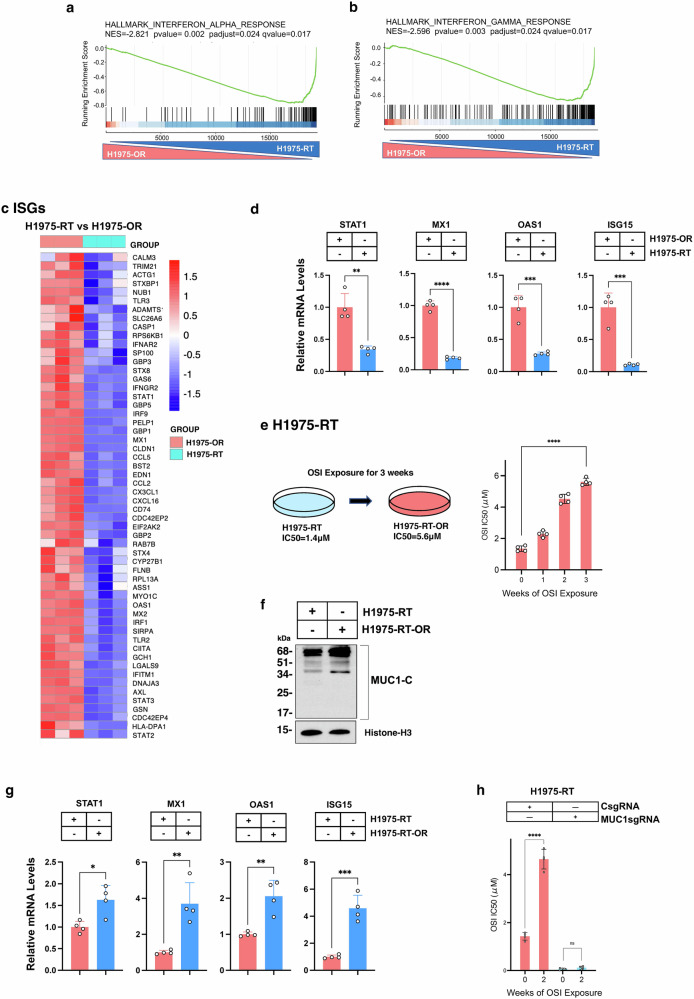
H1975-RT cells are dependent on MUC1-C for activating the memory response of osimertinib resistance. **a**, **b** RNA-seq was performed on biologic triplicates of H1975-OR and H1975-RT cells. GSEA of the H1975-OR vs H1975-RT cell transcriptomes using the indicated HALLMARK gene signatures. **c** Heatmaps of genes downregulated in H1975-RT vs H1975-OR cells. **d** H1975-OR and H1975-RT cells were analyzed for the indicated transcripts by qRT-PCR using primers listed in Supplemental Table 1. The results (mean±SD of 4 determinations) are expressed as relative levels compared to those obtained for H1975-OR cells (assigned a value of 1). **e** H1975-RT cells exposed to osimertinib for 1-3 weeks were analyzed for osimertinib sensitivity by Alamar Blue staining (Supplementary Fig. 2b). The results (mean±SD of 4 determinations) are expressed as the osimertinib IC50 μM values. **f** Chromatin from H1975-RT and H1975-RT-OR cells was immunoblotted with antibodies against the indicated proteins. **g** H1975-RT and H1975-RT-OR cells were analyzed for the indicated transcripts by qRT-PCR. The results (mean±SD of 4 determinations) are expressed as relative levels compared to that obtained for H1975-RT cells (assigned a value of 1). **h** H1975-RT/CsgRNA and H1975-RT/MUC1sgRNA cells exposed to osimertinib for 2 weeks were analyzed for osimertinib sensitivity by Alamar Blue staining (Supplementary Fig. 2c). The results (mean±SD of four determinations) are expressed as the osimertinib IC50 μM values

Targeting MUC1-C in H1975-OR cells and in patient-derived MGH170 and MGH121 cells with acquired resistance to osimertinib reverses the resistant phenotype.^39^ In assessing biological significance of the H1975-RT phenotype, comparison of H1975-RT vs H1975-OR cells revealed significant downregulation of the HALLMARK INTERFERON ALPHA and HALLMARK INTERFERON GAMMA RESPONSE signatures (Fig. 2a and 2b; Supplementary Fig. 2a). Analysis of H1975-RT vs H1975-OR cells also identified decreases in STAT1 and downstream ISGs (Fig. 2c), which were confirmed for selected genes by qRT-PCR (Fig. 2d). These results substantiated that (i) osimertinib resistance is associated with activation of IFN type I/II signaling, and (ii) the H1975-RT phenotype is associated with downregulation of these pathways.

We next exposed H1975-RT cells to osimertinib, which reestablished stable osimertinib-resistant H1975-RT-OR cells with an IC50 = 5.6 μM (Fig. 2e; Supplementary Fig. 2b). Noteworthy is that H1975-RT-OR cells were selected after 3 weeks (Fig. 2e), as compared to 12 weeks for achieving the same IC50 = 5.6 μM in the selection of H1975 to H1975-OR cells. Concordant with reestablishing the osimertinib-resistant phenotype, comparison of H1975-RT-OR vs H1975-RT cells identified increases in (i) MUC1-C chromatin levels (Fig. 2f) and (ii) expression of STAT1, MX1, OAS1, and ISG15 (Fig. 2g).

To assess involvement of MUC1-C in recalling this response, we exposed H1975-RT/MUC1sgRNA cells with MUC1-C silencing to osimertinib and found that the emergence of resistance is abrogated as compared to that in control H1975-RT/CsgRNA cells (Fig. 2h; Supplementary Fig. 2c). In accordance with these results, exposure of PC9/vector cells with osimertinib for 2 weeks had a modest effect on drug sensitivity (OSI IC50 = 0.055 μM; Supplementary Fig. 2d). By contrast and as found for H1975-RT-OR cells, treatment of PC9/MUC1-C cells with osimertinib was associated with the development of resistant PC9/MUC1-C-OR cells after 2 weeks (OSI IC50 = 1.5 μM; Supplementary Fig. 2d). Additionally, like H1975-RT-OR cells, PC9/MUC1-C-OR cells exhibited higher MUC1-C chromatin levels compared to PC9/MUC1-C cells (Supplementary Fig. 2e).

These findings reveal that MUC1-C-dependent regulation of the STAT1 pathway is of importance for (i) establishing TKI resistance in H1975-OR cells, (ii) remembering the response of H1975-RT cells to TKI in H1975-RT-OR cells, and (iii) conferring development of TKI resistance in PC9 cells (Supplementary Fig. 2f).

### Activation of a *MUC1* proximal enhancer-like signature (pELS-1) in the memory of H1975-RT cells to TKI resistance

**Fig. 3:**
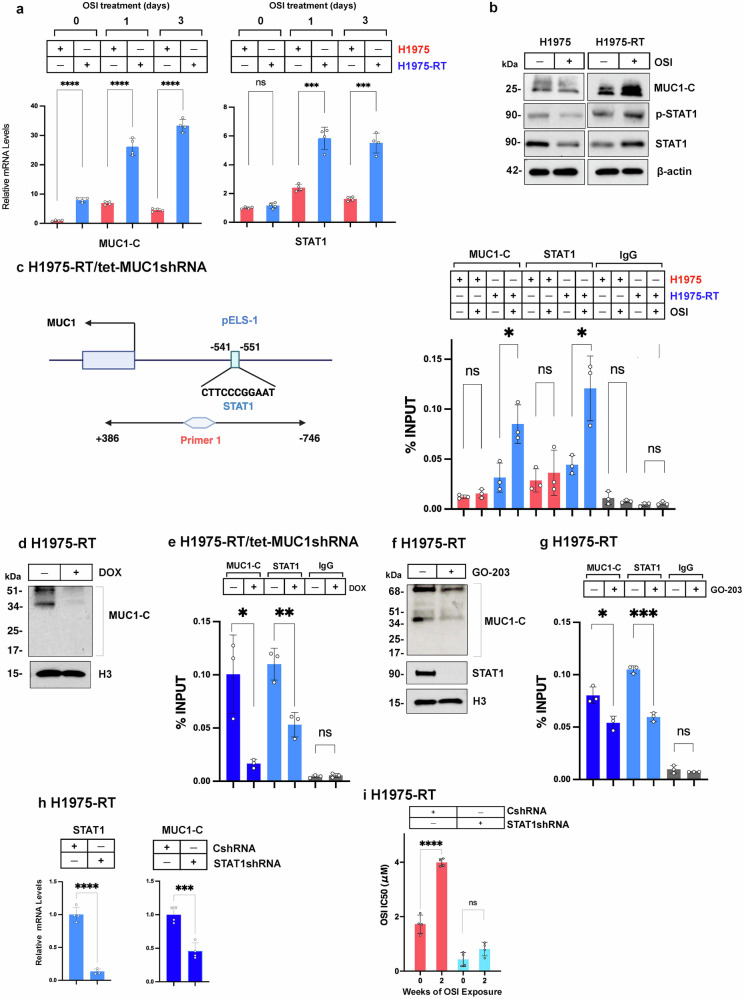
*MUC1* is induced by a STAT1-mediated pathway in the memory response to osimertinib treatment. **a** H1975 and H1975-RT cells treated with 1 μM osimertinib for 1-3 days were analyzed for MUC1-C (left) and STAT1 (right) mRNA levels by qRT-PCR. The results (mean±SD of 4 determinations) are expressed as relative levels compared to that obtained for untreated H1975 cells (assigned a value of 1). **b** Lysates from H1975 and H1975-RT cells treated with 1 μM osimertinib for 2 days were immunoblotted with antibodies against the indicated proteins. **c** Schematic of the *MUC1* gene highlighting a proximal enhancer-like signature-1 (pELS-1) with a STAT1 binding motif. Soluble chromatin from H1975 and H1975-RT cells treated with vehicle or 1 μM osimertinib for 2 days was precipitated with anti-MUC1-C, anti-STAT1 or a control IgG. The DNA samples were amplified by qPCR with primer set-1 encompassing +386 to -746 bp to the TSS (Supplementary Table 2). The results (mean ± SD of 3 determinations) are expressed as percent input. **d**, **e** H1975-RT/tet-MUC1shRNA cells were treated with vehicle or DOX for 7 days. Chromatin was immunoblotted with antibodies against the indicated proteins (**d**) Soluble chromatin was precipitated with anti-MUC1-C, anti-STAT1 or a control IgG (**e**). The DNA samples were amplified by qPCR for pELS-1. The results (mean ± SD of 3 determinations) are expressed as percent input. **f**, **g** H1975-RT cells were treated with vehicle or 3 μM GO-203 for 3 days. Chromatin was immunoblotted with antibodies against the indicated proteins (**f**). Soluble chromatin was precipitated with anti-MUC1-C, anti-STAT1 or IgG (**g**). The DNA samples were amplified by qPCR for pELS-1. The results (mean ± SD of 3 determinations) are expressed as percent input. **h** H1975-RT/CshRNA and H1975-RT/STAT1shRNA cells treated with 1 μM osimertinib for 2 days were analyzed for STAT1 and MUC1-C mRNA levels by qRT-PCR. The results (mean±SD of four determinations) are expressed as relative levels compared to that obtained for H1975-RT/CshRNA cells (assigned a value of 1). **i** H1975-RT/CshRNA and H1975-RT/STAT1shRNA cells exposed to 1 μM osimertinib for 2 weeks were analyzed for osimertinib sensitivity by Alamar Blue staining (Supplementary Fig. 3d). The results (mean±SD of 4 determinations) are expressed as osimertinib IC50 values

In further assessing MUC1-C involvement, we found that exposure of H1975-RT cells to osimertinib induces a rapid ~30-fold upregulation of MUC1-C mRNA levels; whereas, MUC1-C transcripts in osimertinib-treated parental H1975 cells were increased by ~4-fold (Fig. 3a). We also found that increases in STAT1 mRNA levels are more pronounced in osimertinib-treated H1975-RT vs H1975 cells (Fig. 3a). Osimertinib-induced MUC1-C and STAT1 mRNA levels were associated with upregulation of MUC1-C, p-STAT1 and STAT1 proteins in total lysates from H1975-RT cells (Fig. 3b). By contrast, MUC1-C, p-STAT1 and STAT1 protein levels were in part suppressed in osimertinib-treated H1975 cells (Fig. 3b), consistent with differential regulation of MUC1-C and STAT1 expression in the response of H1975-RT and H1975 cells to osimertinib exposure. PC9/MUC1-C cells treated with osimertinib also exhibited higher levels of STAT1 transcripts compared to PC9/vector cells (Supplementary Fig 3a).

MUC1-C binds to STAT1 in regulating STAT1 target genes that include *MUC1* in an auto-inductive pathway.^19^ *MUC1* contains a STAT binding motif in a proximal enhancer-like signature-1 (pELS-1; -541 to -551 bp upstream to the TSS)(Fig. 3c). We found that pELS-1 is occupied by MUC1-C and STAT1 and that their occupancy is significantly increased in control and osimertinib-treated H1975-RT, but not H1975, cells (Fig. 3c). Furthermore, silencing MUC1-C in H1975-RT cells decreased chromatin MUC1-C levels (Fig. 3d) and occupancy of STAT1 on pELS-1 (Fig. 3e). Previous work found that targeting MUC1-C with the GO-203 inhibitor reverses osimertinib resistance;^39^ however, the mechanistic basis for this finding was not addressed in those studies. Here, we found that GO-203 treatment of H1975-RT cells suppresses (i) chromatin MUC1-C and STAT1 levels (Fig. 3f) and (ii) STAT1 occupancy on pELS-1 (Fig. 3g), indicating that MUC1-C is required for localization of STAT1 in chromatin.

This intersection of MUC1-C and STAT1 was extended by the observations that (i) silencing MUC1-C in H1975-RT cells suppresses STAT1 expression (Supplementary Fig. 3b), and (ii) silencing STAT1 downregulates MUC1-C expression (Fig. 3h; Supplementary Fig. 3c). Moreover, like MUC1-C, STAT1 was necessary for reestablishing osimertinib resistance (Fig. 3i; Supplementary Fig. 3d). In support of the recognized crosstalk between STAT1 and STAT2, other unpublished studies have demonstrated that silencing STAT2 in H1975-RT cells downregulates STAT1 and MUC1-C expression. These results in osimertinib-treated H1975-RT cells support activation of an inflammatory memory response by the auto-inductive MUC1-C/STAT1 pathway that drives ISG expression and promotes osimertinib resistance (Supplementary Fig. 3e).

### AP-1 signaling activates a *MUC1* proximal enhancer-like signature-2 (pELS-2) in the memory of H1975-RT cells to TKI resistance

**Fig. 4:**
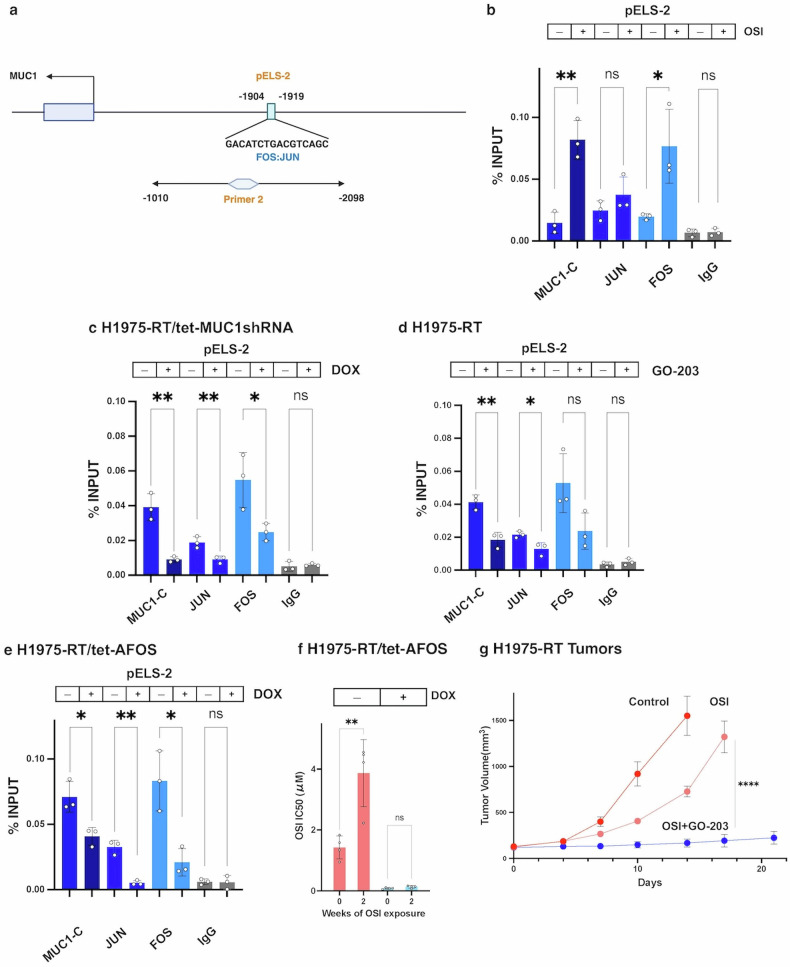
Induction of MUC1-C in the inflammatory memory response to osimertinib treatment is JUN/AP-1 dependent. **a** Schematic of the *MUC1* gene highlighting a FOS:JUN binding motif in pELS-2. **b** Soluble chromatin from H1975-RT cells was precipitated with anti-MUC1-C, anti-JUN, anti-FOS or a control IgG. The DNA samples were amplified by qPCR with primer set-2 encompassing −1010 to −2098 bp to the TSS (Supplemental Table 2). The results (mean ± SD of 3 determinations) are expressed as percent input. **c** Soluble chromatin from H1975-RT/tet-MUC1shRNA cells treated with vehicle or DOX for 7 days and then with 1 μM osimertinib for 2 days was precipitated with antibodies against the indicated proteins and a control IgG. The DNA samples were amplified by qPCR for pELS-2. The results (mean ± SD of 3 determinations) are expressed as percent input. **d** Soluble chromatin from H1975-RT cells treated with 1 μM osimertinib and vehicle or 3 μM GO-203 for 2 days was precipitated with antibodies against the indicated proteins and a control IgG. The DNA samples were amplified by qPCR for pELS-2. The results (mean ± SD of 3 determinations) are expressed as percent input. **e** Soluble chromatin from H1975-RT/tet-AFOS cells treated with vehicle or DOX for 7 days and then with 1 μM osimertinib for 2 days was precipitated with antibodies against the indicated proteins and a control IgG. The DNA samples were amplified by qPCR for pELS-2. The results (mean ± SD of 3 determinations) are expressed as percent input. **f** H1975-RT/tet-AFOS cells treated with vehicle or DOX for 7 days and then exposed to 1 μM osimertinib for 2 weeks were analyzed for osimertinib sensitivity by Alamar Blue staining (Supplementary Fig. 4d). The results (mean±SD of 4 determinations) are expressed as osimertinib IC50 μM values. **g** Six-week old nude mice were injected subcutaneously in the flank with 5 × 10^6^ H1975-RT cells. Mice were pair-matched into three groups of 5 mice each when tumors reached 100–150 mm^3^ that were treated with vehicle control (red), osimertinib (orange), and GO-203 plus osimertinib (blue) for the indicated days. Tumor volumes are expressed as the mean±SEM for five mice

MUC1-C interacts directly with JUN and regulates the expression of FOS, which forms complexes with JUN on enhancers.^29,45^ *MUC1* contains a pELS-2 region for putative JUN/AP-1 binding (-1904 to -1919 bp from the TSS)(Fig. 4a). Treatment of H1975-RT cells with osimertinib significantly increased occupancy of pELS-2 by MUC1-C and FOS, but not JUN (Fig. 4b). By comparison, osimertinib had no significant effect on JUN or FOS occupancy of pELS-2 in H1975 cells (Supplementary Fig. 4a). Silencing MUC1-C in H1975-RT cells inhibited osimertinib-induced upregulation of MUC1-C and AP-1 occupancy on pELS-2 (Fig. 4c). Similar suppressive effects were observed by targeting MUC1-C function with the GO-203 inhibitor (Fig. 4d), indicating that MUC1-C is necessary for osimertinib-induced increases in pELS-2 occupancy by AP-1.

We therefore targeted AP-1 with a tet-inducible AFOS dominant-negative vector that disrupts binding of FOS and JUN to DNA.^45^ DOX treatment of H1975-RT/tet-AFOS cells significantly suppressed pELS-2 occupancy by MUC1-C, JUN and FOS (Fig. 4e). Targeting JUN/AP-1 with AFOS also (i) inhibited osimertinib-induced expression of MUC1-C, STAT1 and ISGs (Supplementary Figs. 4b and 4c), and (ii) blocked the memory of osimertinib resistance (Fig. 4f; Supplementary Fig. 4d). Targeting MUC1-C with silencing or GO-203 in H1975-RT cells similarly inhibited osimertinib-driven induction of MUC1-C, STAT1, ISG and FOS expression (Supplementary Fig. 4e and 4f). In concert with these results, PC9/MUC1-C cells treated with osimertinib exhibited upregulation of MX1, OAS1, and ISG15 compared to PC9 cells (Supplementary Fig. 4g).

To extend these findings, we treated H1975-RT tumor xenografts in mice with osimertinib alone and in combination with GO-203 to determine if targeting MUC1-C has an effect on the osimertinib-resistant phenotype in vivo. We found that osimertinib alone has a partial inhibitory effect on growth and, as shown for suppression of MUC1-C signaling in vitro, GO-203 is effective against osimertinib-resistant tumors (Fig. 4g). These results supported activation of the *MUC1* pELS-2 in recalling an AP-1-mediated response to TKI resistance (Supplementary Fig. 4h).

### Chromatin accessibility of the *MUC1* pELS domains is regulated by PBRM1/PBAF

**Fig. 5:**
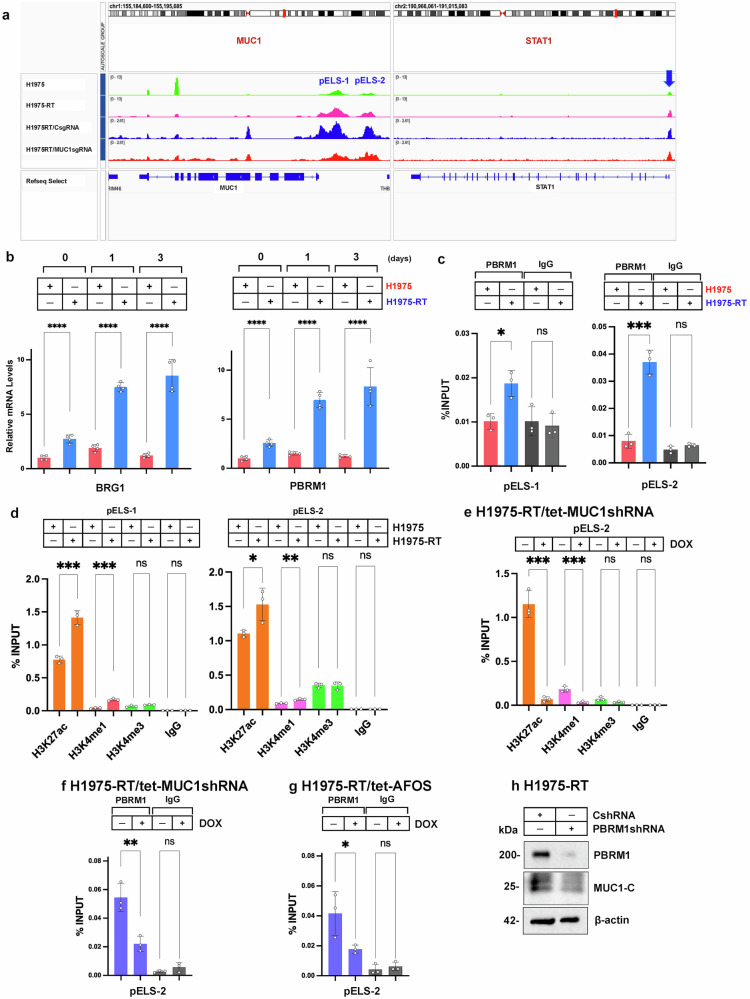
MUC1-C expression induced in the inflammatory memory response to osimertinib treatment is PBRM1/PBAF dependent. **a** ATAC-seq was performed in (i) triplicates on H1975 and H1975-RT cells, and (ii) duplicates on H1975-RT/CsgRNA and H1975-RT/MUC1sgRNA cells. Representative genome browser snapshots are shown for the *MUC1* and *STAT1* genes. Highlighted are the *MUC1* pELS-1 (-541 to -551 bp upstream to the TSS) and pELS-2 (-1904 to -1909 bp upstream to the TSS) regions. Also highlighted is the *STAT1* promoter region that is activated by an auto-inductive STAT1 binding motif. **b** H1975 and H1975-RT cells treated with 1 μM osimertinib for 1-3 days were analyzed for BRG1 (left) and PBRM1 (right) mRNA levels by qRT-PCR. The results (mean±SD of 4 determinations) are expressed as relative levels compared to that obtained for untreated H1975 cells (assigned a value of 1). **c** Soluble chromatin from H1975 and H1975-RT cells treated with vehicle or 1 μM osimertinib for 2 days was precipitated with anti-PBRM1 or IgG. The DNA samples were amplified by qPCR for pELS-1 and pELS-2. The results (mean ± SD of 3 determinations) are expressed as percent input. **d** Soluble chromatin from H1975 and H1975-RT cells was precipitated with antibodies against the indicated proteins and a control IgG. The DNA samples were amplified by qPCR for pELS-1 and pELS-2. The results (mean ± SD of 3 determinations) are expressed as percent input. **e** Soluble chromatin from H1975-RT/tet-MUC1shRNA cells treated with vehicle or DOX for 7 days was precipitated with antibodies against the indicated proteins and a control IgG. The DNA samples were amplified by qPCR for pELS-2. The results (mean ± SD of 3 determinations) are expressed as percent input. **f** H1975-RT/tet-MUC1shRNA cells were treated with vehicle or DOX for 7 days. Soluble chromatin was precipitated with anti-PBRM1 or IgG. The DNA samples were amplified by qPCR for pELS-2. The results (mean ± SD of 3 determinations) are expressed as percent input. **g** H1975-RT/tet-AFOS cells were treated with vehicle or DOX for 7 days. Chromatin was precipitated with anti-PBRM1 or IgG. The DNA samples were amplified by qPCR for pELS-2. The results (mean ± SD of 3 determinations) are expressed as percent input. **h** Lysates from H1975-RT/CshRNA and H1975/PBRM1shRNA cells treated with 1 μM osimertinib for 2 days were immunoblotted with antibodies against the indicated proteins

Memory domains exhibit increases in chromatin accessibility to rapidly recall exposures to a previous insult.^46^ To assess involvement of MUC1 pELS-1 and pELS-2 in the memory response to osimertinib treatment, we performed ATAC-seq and found that chromatin accessibility of these domains is increased in H1975-RT, as compared to parental H1975, cells (Fig. 5a). We also found that silencing MUC1-C in H1975-RT cells decreases accessibility of the MUC1 pELS-1 and pELS-2 domains (Fig. 5a). Analysis of *STAT1* identified a comparable pattern (Fig. 5a); that is, increased chromatin accessibility of the promoter, rather than proximal enhancer, region in H1975-RT vs H1975 cells by a MUC1-C-mediated mechanism. Further analysis of MUC1-C-induced ISGs in H1975-RT cells uncovered MUC1-C-dependent increases in accessibility of the *IRF1* and *ISG15* promoters (Supplementary Fig. 5a); whereas, in contrast, definitive promoter or proximal enhancer regions with chromatin accessibility were undetectable in the *MX1* and *OAS1* genes (Supplementary Fig. 5a). These results indicate that (i) MUC1-C induces STAT1 and certain downstream ISGs as effectors of the memory response, and (ii) not all MUC1-C-driven ISGs function as inflammatory memory genes. Subsequent experimentation will be needed to determine if MUC1-C induces the *MX1* and *OAS1* genes by an alternative mechanism involving epigenetic transcriptional memory involving bivalent histone modifications.

As potential effectors of the MUC1-C memory response, JUN/AP-1 recruit SWI/SNF chromatin remodeling complexes to enhancers.^47–50^ MUC1-C activates the (i) BAF complex in driving stemness genes, and (ii) PBAF complex in regulating stress-associated genes.^28–30^ GSEA demonstrated significant upregulation of the GOCC SWI/SNF Complex gene signature in H1975-OR vs H1975 cells (Supplementary Fig. 5b). Silencing MUC1-C in H1975-OR cells suppressed this signature (Supplementary Fig. 5c), supporting a potential role for SWI/SNF in the osimertinib memory response. Consistent with these observations, osimertinib treatment of H1975-RT vs H1975 cells increased expression of BRG1 and PBRM1 (Fig. 5b; Supplementary Fig. 5d), but not ARID1A (Supplementary Fig. 5e), indicating involvement of PBRM1/PBAF. MUC1-C dependency was substantiated by the demonstration that silencing MUC1-C in H1975-RT cells (Supplementary Fig. 5f) and GO-203 treatment (Supplementary Fig. 5g) suppresses osimertinib-induced increases in BRG1 and PBRM1 expression. Consistent with these results, studies of PC9/MUC1-C vs PC9/vector cells confirmed upregulation of BRG1 and PBRM1 (Supplementary Fig. 5h). Further analysis demonstrated that PBRM1 occupancy of the *MUC1* pELS-1 and pELS-2 regions is increased in H1975-RT vs H1975 cells (Fig. 5c).

Memory domains are associated with modifications of H3K27 acetylation and H3K4 methylation patterns.^41,46,51^ Along these lines, we found that H3K27ac and H3K4me1, but not H3K4me3, levels on pELS-1 and pELS-2 are increased in H1975-RT vs H1975 cells (Fig. 5d). In focusing on pELS-2, silencing MUC1-C in H1975-RT cells decreased (i) H3K27ac and H3K4me1 deposition (Fig. 5e), and (ii) PBRM1 occupancy (Fig. 5f). Targeting JUN/AP-1 with AFOS also decreased PBRM1 occupancy on pELS-2 (Fig. 5g). Furthermore, silencing PBRM1 suppressed MUC1-C expression (Fig. 5h) and increased sensitivity to osimertinib-induced loss of clonogenic survival (Supplementary Fig. 5i). These results indicate that the *MUC1* pELS-1/2 regions drive inflammatory memory by MUC1-C-induced regulation of (i) chromatin accessibility, (ii) PBAF/PBRM1-mediated chromatin remodeling, and (iii) H3K27ac and H3K4me1 deposition (Supplementary Fig. 5j).

### MUC1-C drives an inflammatory memory pathway in NSCLC cells derived from patients with acquired TKI resistance

**Fig. 6:**
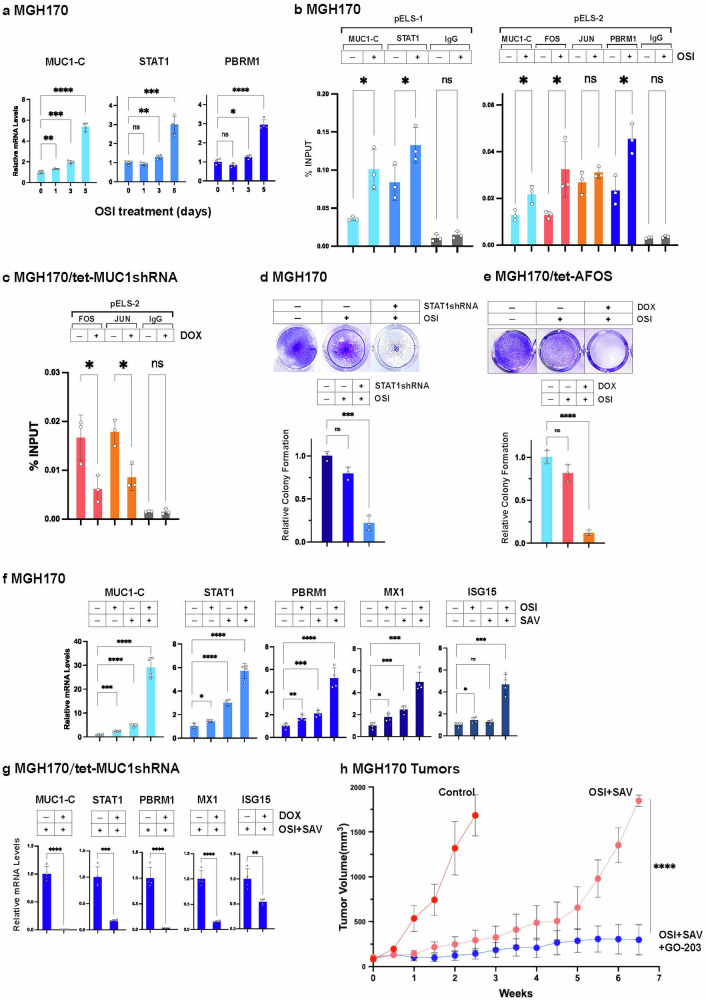
MET-amplified MGH170 cells are dependent on MUC1-C for MET inhibitor resistance and survival. **a** MGH170 cells treated with 1 μM osimertinib for the indicated days were analyzed for MUC1-C, STAT1 and PBRM1 mRNA levels by qRT-PCR. The results (mean±SD of four determinations) are expressed as relative levels compared to that obtained for untreated cells (assigned a value of 1). **b** Soluble chromatin from MGH170 cells treated with vehicle or 1 μM osimertinib for 3 days was precipitated with antibodies against the indicated proteins or a control IgG. The DNA samples were amplified by qPCR for pELS-1 and pELS-2. The results (mean ± SD of 3 determinations) are expressed as percent input. **c** Soluble chromatin from MGH170/tet-MUC1shRNA cells treated with vehicle or DOX and 1 μM osimertinib for 3 days was precipitated with antibodies against the indicated proteins or a control IgG. The DNA samples were amplified by qPCR for pELS-2. The results (mean ± SD of 3 determinations) are expressed as percent input. **d** MGH170/CshRNA and MGH170/STAT1shRNA cells treated with 1 μM osimertinib were analyzed for clonogenic survival. Shown are representative photomicrographs of stained colonies. The results (mean±SD of three determinations) are expressed as relative colony formation compared to that for untreated cells (assigned a value of 1). **e** MGH170/tet-AFOS cells treated with vehicle or DOX for 14 days were analyzed for clonogenic survival. Shown are representative photomicrographs of stained colonies. The results (mean±SD of three determinations) are expressed as relative colony formation compared to that for untreated cells (assigned a value of 1). **f** MGH170 cells treated with 0.5 μM osimertinib and/or 0.5 μM savolitinib for 2 days were analyzed for the indicated mRNA levels by qRT-PCR. The results (mean±SD of 4 determinations) are expressed as relative levels compared to that obtained for untreated cells (assigned a value of 1). **g** MGH170/tet-MUC1shRNA cells treated with vehicle or DOX for 7 days and then with 0.5 μM osimertinib and/or 0.5 μM savolitinib for 2 days were analyzed for the indicated mRNA levels by qRT-PCR. The results (mean±SD of 4 determinations) are expressed as relative levels compared to that obtained for untreated cells (assigned a value of 1). **h** Six-week old nude mice were injected subcutaneously in the flank with 5 × 10^6^ MGH170 cells. Mice pair-matched into three groups of 5 mice each when tumors reached 100–150 mm^3^ were treated with vehicle control (red), OSI + SAV (orange), and OSI + SAV plus GO-203 (blue) for the indicated days. Tumor volumes are expressed as the mean±SEM for five mice

We next focused on MGH170-1D #2 (MGH170) cells with *MET* amplification and acquired osimertinib resistance.^52^ As found in H1975-RT cells, osimertinib treatment of MGH170 cells was associated with induction of MUC1-C, STAT1 and PBRM1 expression (Fig. 6a). Osimertinib increased (i) MUC1-C and STAT1 occupancy on the *MUC1* pELS-1 and (ii) MUC1-C, FOS and PBRM1, but not JUN, occupancy on pELS-2 (Fig. 6b). Silencing MUC1-C decreased osimertinib-induced (ii) JUN and FOS occupancy on *MUC1* pELS-2 (Fig. 6c). Silencing MUC1-C in MGH170 cells also suppressed STAT1 and ISG expression (Supplementary Fig. 6a). Additionally, like MUC1-C,^39^ targeting STAT1 with silencing (Fig. 6d) and JUN/AP-1 with AFOS (Fig. 6e) sensitized MGH170 cells to osimertinib treatment, providing further evidence for activation of the *MUC1* pELS-1/2 regions in the memory response to osimertinib resistance.

Approximately 25% of patients who progress on osimertinib develop *MET* amplification or MET-associated resistance mechanisms.^11^ Accordingly, osimertinib is being combined with MET inhibitors, such as capmatinib and savolitinib, to circumvent this mechanism of osimertinib resistance.^53^ Nonetheless, patients invariably develop resistance to combined treatment with osimertinib+MET inhibitors.^54^ As expected, treatment with capmatinib or savolitinib sensitized MGH170 cells to osimertinib (Supplementary Fig. 6b). Treatment of MGH170 cells with osimertinib in combination with these MET inhibitors increased expression of the MUC1-C-driven STAT1, PBRM1 and ISG pathway (Fig. 6f; Supplementary Fig. 6c). Combining osimertinib with capmatinib or savolitinib, further increased expression of MUC1-C, STAT1, PBRM1 and downstream ISGs (Fig. 6f; Supplementary Fig. 6c). Moreover, silencing MUC1-C in MGH170 cells suppressed these responses (Fig. 6g; Supplementary Fig. 6d). Treatment of MGH170 cells with GO-203 also suppressed expression of MUC1-C, STAT1, PBRM1 and ISG (Supplementary Fig. 6e). Prior studies found that treatment of MGH170 tumor xenografts with GO-203 suppresses their growth, but they did not address whether this MUC1-C inhibitor has an effect in the setting of TKI treatment resistance.^39^ We therefore treated mice bearing established MGH170 tumor xenografts with osimertinib+savolitinib alone and in combination with GO-203 to determine if targeting MUC1-C has an effect on the osimertinib+savolitinib resistant phenotype in vivo. The results demonstrated that MGH170 xenografts exhibit resistance to osimertinib+savolitinib treatment and that targeting MUC1-C with GO-203 is effective against the resistant tumors (Fig. 6h).

The *EGFR* C797S mutation confers resistance to osimertinib as the next most frequent mechanism after *MET* amplification.^55,56^ Patient-derived MGH121 Res#2 cells harbor the EGFR(T790M/C797S) mutation and are resistant to osimertinib (IC50 = 4.6 μM).^57^ Osimertinib treatment of MGH121 cells resulted in modest upregulation of MUC1-C, STAT1, and ISG expression; whereas, intriguingly, more pronounced increases were observed with the 4^th^ generation EGFR-TKI TQB3804 (Supplementary Fig. 6f), which is active against the T790M/C797S compound mutation.^58,59^ Targeting MUC1-C with GO-203 treatment of MGH121 cells attenuated TQB3804-induced upregulation of the MUC1-C/STAT1 pathway (Supplementary Fig. 6f) and enhanced sensitivity to TQB3804 (Supplementary Fig. 6g). These findings in patient-derived NSCLC cells supported MUC1-C dependence for activation of a memory pathway that is mediated by STAT1 and drives resistance to osimertinib and other TKI inhibitors.

### MUC1-C is a target for M1C ADC treatment of patients with TKI-resistant EGFR mutant NSCLCs

Analysis of the TCGA LUAD database previously found that patients with EGFR mutant NSCLCs expressing high vs low MUC1 levels have a significantly shorter overall survival.^15^ These findings were limited to EGFR mutant NSCLCs in that MUC1 levels are not associated with survival in patients with EGFR wild-type NSCLCs. The basis for this distinction is unclear and will require further study of MUC1-C protein levels in NSCLCs with wild-type EGFR. Along these lines, analysis of EGFR mutant NSCLCs has demonstrated that patients with high MUC1-C protein levels in their tumors experience a decrease in recurrence-free survival after surgical resection,^39^ indicating that MUC1-C is a target for the treatment of EGFR mutant NSCLCs.

**Fig. 7:**
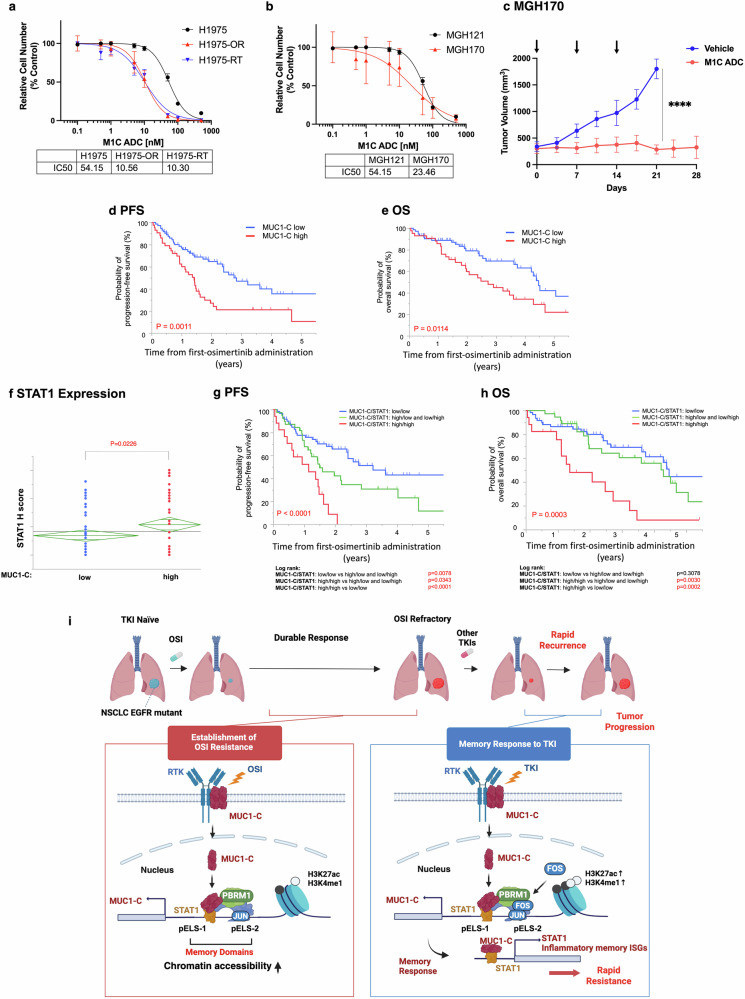
MUC1-C expression in EGFR mutant NSCLCs associates with response to osimertinib. The designated NSCLC cell lines (**a**) and patient-derived cells (**b**) were treated with the indicated concentrations of M1C ADC for 7 days and analyzed for cell viability by Alamar Blue staining. The results (mean±SD of six determinations) are expressed as relative cell viability (% control). Indicated are the anti-MUC1-C ADC IC50 values. **c** Mice bearing established MGH170 PDX tumors were pair-matched into two groups of six mice each when tumors reached 100 mm^3^. Control vehicle or M1C ADC (7.5 mg/kg) was administered intravenously in the tail vein on the indicated days. Tumor volumes are expressed as the mean ± SD. Kaplan–Meier curves for PFS (**d**) and OS (**e**) of patients with EGFR mutant NSCLCs treated with osimertinib according to high (red) or low (blue) levels of MUC1-C expression. P values were obtained using the log rank test. **f** Association of MUC1-C low and high levels with STAT1 expression. Kaplan–Meier curves for PFS (**g**) and OS (**h**) of patients with EGFR mutant NSCLCs treated with osimertinib according to (i) MUC1-C/STAT1 low/low (blue), (ii) MUC1-C/STAT1 high/low or low/high (green), and (iii) MUC1-C/STAT1 high/high (red) levels of expression. **i** Schemas depicting the MUC1-C-driven inflammatory memory response that confers resistance of NSCLC cells to osimertinib. MUC1-C homodimers form complexes with EGFR and other RTKs, such as MET, at the NSCLC cell membrane. In establishing TKI resistance (left), targeting EGFR with osimertinib induces MUC1-C-mediated activation of the *MUC1* gene in an auto-inductive pathway. This response to osimertinib includes occupancy of *MUC1* memory domains (i) pELS-1 by MUC1-C and STAT1, and (ii) pELS-2 by MUC1-C, AP-1 (JUN) and PBAF (BRG1, PBRM1). In turn, upregulation of MUC1-C induces *STAT1* and thereby the formation of MUC1-C/STAT1 complexes that regulate the expression of downstream ISGs in association with DNA damage resistance and immune evasion.^25–27,73–75^ In conferring the memory response after a drug holiday (right), the *MUC1* pELS-1 and pELS-2 domains exhibit increases in (i) chromatin accessibility and (ii) H3K27ac and H3K4me1 deposition. In this way, cells are poised to respond to subsequent treatment with osimertinib, as well as MET-TKIs and the EGFR-TKI TQB3804, with rapid induction of (i) MUC1-C, STAT1 and the IFN type I/II pathways, and (ii) recurrence of refractory disease. The findings that targeting MUC1-C genetically or pharmacologically suppresses this memory response of TKI resistance have uncovered MUC1-C as a target for M1C ADC, as well as small molecule inhibitor, treatment of NSCLC patients with refractory tumors. Images generated using BioRender software

Targeting MUC1-C with an antibody-drug conjugate (M1C ADC) is effective against NSCLC HCC827 cells with an activating EGFR(del E746-A750) mutation sensitive to TKIs growing in vitro and as tumor xenografts.^60^ However, it is not known if the M1C ADC is active in settings of NSCLC TKI resistance. In addressing this issue of translational importance, we found that H1975-OR, as well as H1975-RT, cells are sensitive to the M1C ADC with IC50s in the nM range, which are comparable to parental H1975 cells (Fig. 7a). We also found that TKI-resistant MGH170 and MGH121 cells are sensitive to the M1C ADC with nM IC50s (Fig. 7b), indicating that this ADC could be effective against NSCLCs dependent on MUC1-C for TKI resistance. In support of this premise, the M1C ADC was highly effective in the treatment of established MGH170 PDX tumor xenografts (Fig. 7c) in the absence of significant weight loss (Supplementary Fig. 7a).

Given the potential importance of MUC1-C as a target for TKI-resistant NSCLCs, we analyzed MUC1-C expression by immunohistochemistry (IHC) in biopsied tumors from a cohort of 116 patients who subsequently received osimertinib therapy. As expected, chromatin-bound proteins were not detectable by IHC of formalin-fixed paraffin-embedded (FFPE) patient tumor samples. Of the 116 tumors, 109 (93.9%) showed detectable levels of MUC1-C expression in the cell membrane and cytoplasm (Supplementary Fig. 7b), which formed the basis for distinguishing between MUC1-C-low vs -high levels. Using the Allred score, we identified 73 tumors (63%) with low MUC1-C levels and 43 tumors (37%) with high MUC1-C levels that were separated according to patient characteristics and clinicopathologic features (Supplementary Fig. 7c). Patients with high vs low cytoplasmic MUC1-C expression in their tumors had significantly shorter progression-free survival (PFS; p = 0.0011) and overall survival (OS; *p* = 0.0114) (Fig. 7d and e). Univariate analyses further showed that MUC1-C high vs low expression is the single variable that significantly associates with decreases in both PFS and OS (Supplementary Fig. 7d). In multivariate analyses, high MUC1-C levels were further identified as independent prognostic factors for PFS and OS (Supplementary Fig. 7e).

We also analyzed STAT1 expression, which, like MUC1-C, identified detectable levels in the cytoplasm (Supplementary Fig. 7f). Consistent with our in vitro studies, high cytoplasmic expression of STAT1 significantly correlated with high MUC1-C cytoplasmic expression (Fig. 7f). High expression of either MUC1-C or STAT1 was significantly associated with shorter PFS compared to their low expression (Fig. 7g). Furthermore, MUC1-C/STAT1 high/high levels were significantly associated with shorter PFS (Fig. 7g) and OS (Fig. 7h).

## Discussion

Plasticity of barrier tissue stem cells enables reprogramming after damage for the restoration of homeostasis and tissue regeneration.^61,62^ Plasticity of cancer cells, which has been exploited from normal tissue stem cells, endows the capacity to overcome the selective pressures in response to treatment.^63^ *MUC1* emerged in mammals to protect barrier tissues from biotic and abiotic insults by regulating plasticity and inflammatory remodeling necessary for wound repair.^17,18^ These responses are theoretically reversible; however, prolonged MUC1-C activation, which regulates epigenetic reprogramming, irreversibly promotes plasticity in cancer progression.^18^ The present work uncovers a role for MUC1-C in conferring memory of cancer cells to treatment. We demonstrate that MUC1-C establishes resistance of NSCLC cells to osimertinib by activating the inflammatory STAT1 and IFN type I/II pathways. In this way, MUC1-C drives expression of ISGs that evolved for protection against viruses, but have been adapted by cancer cells to confer DNA damage resistance and immune evasion.^37,38,40,41,64^ Our results show that MUC1-C is essential for activating this response to osimertinib treatment and, importantly, thereby for establishing the osimertinib-resistant phenotype. Of further significance, studies of osimertinib-resistant cells selected after a drug holiday with a sensitive phenotype retain dependence on MUC1-C for memory to treatment with this agent. Memory of previous insults enables barrier tissues to more rapidly and effectively inform responses to subsequent exposures.^36,46^ We show that the *MUC1* gene responds to osimertinib with activation of pELS regions that function as memory domains. We find that the *MUC1* (i) pELS-1 responds to osimertinib with occupancy of MUC1-C and STAT1, and (ii) pELS-2 acts as a region for increased occupancy of MUC1-C JUN/AP-1 and PBRM1 (Fig. 7i). In accordance with these results, targeting MUC1-C, STAT1, JUN/AP-1 or PBRM1 attenuated the memory response to TKI resistance.

The MUC1-C/STAT1 driven inflammatory pathway could arguably represent an adaptation of barrier tissue stem cells to loss of homeostasis that has been subverted as a maladaptation to protect against osimertinib treatment. Memory of mammalian barrier tissues has been recognized as involving cooperative functions ascribed to epithelial, immune, neuronal, and stromal cells, among others.^65–67^ Studies of epidermal stem cells have shown that the inflammatory memory response to imiquimod encompasses activation of STAT3 as a stimulus-specific inflammatory response and formation of JUN/FOS complexes to maintain memory.^46^ The present results mirror those findings by identifying STAT1 as an osimertinib stimulus-selective inflammatory response in NSCLC cells. Chronic activation of STAT1 was also identified in cancer cells with acquired epigenetic features of inflammatory memory and resistance to immune checkpoint blockade.^41^ As found here, those studies identified dependence on JUN/AP-1 for maintenance and memory.^41^ Of interest in that regard, studies of mouse Lewis Lung Carcinoma (LLC) NSCLC cells that express human MUC1-C (LLC/MUC1) and grow in immunocompetent MUC1 transgenic mice have demonstrated that MUC1-C confers upregulation of PD-L1 in tumors and resistance to immune checkpoint inhibitors.^68,69^ Another common factor associated with memory in barrier tissue stem cells and cancer cells is the reprogramming of H3K27ac and H3K4me1/3 modifications at memory domains.^41,46^ Increases in H3K27ac and H3K4me1 deposition in the response to inflammation are retained at memory domains.^46^ These modifications were identified in the present work for H3K27ac and H3K4me1 at the *MUC*1 pELS regions, in support of their involvement as *MUC1* gene memory domains (Fig. 7i). The novelty here is the critical involvement of MUC1-C in recruitment of STAT1, JUN/AP-1 and PBAF to the *MUC*1 pELS memory domains in a MUC1-C/STAT1 auto-regulatory pathway (Fig. 7i). Subsequent studies will determine if MUC1-C is of importance in other settings of inflammatory memory and whether this pathway is linked to the induction of other ISGs, such as the A3s, that confer treatment resistance.^24,41,46^

Acquired osimertinib resistance in NSCLC cells is frequently associated with *MET* gene amplification and acquisition of the secondary *EGFR* C797S mutation.^11,56^ The present results lend credence to those observations by demonstrating that the MUC1-C-dependent inflammatory response to osimertinib resistance is activated in patient-derived (i) MGH170 cells with *MET* gene amplification, and (ii) MGH121/EGFR(T790M/C797S) cells. Of interest, targeting MET with capmatinib or savolitinib alone or in combination with osimertinib in MGH170 cells also activated the MUC1-C, STAT1, and ISG pathway. Resistance of MGH170 cells to the osimertinib+MET inhibitor combination was abrogated by targeting MUC1-C, indicating that the MUC1-C-driven inflammatory memory pathway is shared in responses to different RTK inhibitors. In NSCLC patients with EGFR-TKI resistance associated with *MET* amplification, combining EGFR-TKIs with a MET inhibitor has shown benefit in several trials.^54,70,71^ The present work demonstrates that, in addition to osimertinib, the MUC1-C inflammatory response confers resistance to MET TKIs and the combination of osimertinib+MET TKIs. We also found that patient-derived MGH121/EGFR(T790M/C797S) cells, which, like MGH170 cells, are dependent on MUC1-C for osimertinib resistance,^39^ respond to treatment with the 4^th^ generation EGFR-TKI TQB3804 by activating the MUC1-C/STAT1 inflammatory pathway. The novelty of these findings is that they have identified MUC1-C as an unanticipated effector of a NSCLC STAT1-mediated inflammatory memory pathway that drives resistance to treatment with (i) combinations of osimertinib+MET TKIs, and (ii) a 4^th^ generation TKI targeting NSCLC cells with the EGFR(T790M/C797S) compound mutation.

Clinical experience with EGFR-TKIs has demonstrated that resistance to the first- and second-generation inhibitors limits responsiveness to treatment with third-generation agents, such as osimertinib.^2,3,72–75^ The basis for this cross-resistance has remained unclear. Our study provides the first evidence that MUC1-C is responsible for the development of osimertinib-resistant NSCLC cells and for memory of that resistance (Fig. 7i). In this regard, the MUC1-C-mediated inflammatory memory pathway provides a plausible explanation for why retreatment with TKIs is invariably ineffective in sustaining responses. Our findings thus shed new light on the basis for this widely recognized outcome of rapidly reacquiring resistance after a drug holiday (Fig. 7i). Patients with EGFR mutant NSCLCs resistant to EGFR-TKIs have limited treatment options. Our results further reveal that the M1C ADC is active against TKI-resistant NSCLC cells in vitro and in a PDX model. These findings and the evidence that expression of MUC1-C in osimertinib-treated EGFR-mutant NSCLCs confers a poor prognosis indicate that the M1C ADC could be effective in abrogating MUC1-C-driven inflammatory memory in patients with NSCLCs resistant to EGFR-TKIs.

## Materials and Methods

### Cell culture

H1975/EGFR(L858R/T790M) and PC9/EGFR(19del) cells (ATCC) were grown in RPMI1640 medium (Thermo Fisher Scientific, Waltham, MA, USA) with 10% fetal bovine serum (FBS; GEMINI Bio-Products, West Sacramento, CA, USA). MGH170-1D #2 (*MET* amplification) and MGH121 Res#2/EGFR(C797S/T790M/19del) cells^52,57,75,76^ were cultured as described.^39^ Cells were treated with osimertinib, GO-203, capmatinib, savolitinib, and TQB3804 (Selleck Chemicals, Houston, TX, USA). Resistant cells maintained in the presence of the drug were grown in drug-free medium for 24 h before analysis. Mycoplasma contamination was monitored every 3-4 months using the MycoAlert Mycoplasma Detection Kit (Lonza, Rockland, MA, USA). Cells were maintained for 3 months when performing experiments and authenticated by STR analysis.

### Gene silencing

MUC1shRNA (MISSION shRNA TRCN0000122938; Sigma, St. Louis, MO, USA) or a control scrambled shRNA (CshRNA; Sigma) was inserted into the pLKO-tet-puro vector (Plasmid #21915; Addgene, Cambridge, MA, USA) as described.^39^ Single guide sgRNAs targeting MUC1 exon 4 were inserted into the lentiCRISPR v2 (Plasmid #52961; Addgene). Vectors were produced in HEK293T cells as described.^39,77^

### Immunoblot analysis

Immunoblot analysis was performed as described^39^ using anti-MUC1-C (#MA5–11202, 1:1000 dilution; Thermo Fisher Scientific), anti-β-actin (A5441, 1:5000 dilution; Sigma-Aldrich, Burlington, MA, USA), anti-p-STAT1 (#9167, 1:1000 dilution; CST), anti-STAT1 (#9172, 1:1000 dilution; CST), anti-OAS1 (#14498, 1:1000 dilution; CST), anti-MX1 (37849, 1:1000 dilution; CST), anti-ISG15 (sc-166755, 1:250 dilution; Santa Cruz, Santa Cruz Biotechnology), anti-PBRM1 (A301–591 A, 1:10000 dilution; Bethyl Laboratories), anti-BRG1 (49360 1:1000 dilution; CST), anti-FOS (#2250, 1:500 dilution; CST) and anti-JUN (#3270 1:1000 dilution; CST) as described.^39^ Chromatin proteins isolated using the chromatin extraction kit (ab117152, Abcam) according to the manufacturer’s instructions were immunoblotted with anti-MUC1-C (#MA5–11202, 1:1000 dilution; Thermo Fisher Scientific) and anti-histone H3 (ab1791, 1:1000 dilution; Abcam).

### Quantitative reverse-transcription PCR (qRT-PCR)

Total cellular RNA was analyzed by qRT-PCR as described.^39^ Primers used for qRT-PCR are listed in Supplementary Table 1.

### Colony formation assays

Cells were analyzed for clonogenic survival as described.^39^

### Chromatin immunoprecipitation

Chromatin immunoprecipitation (ChIP) was performed using a control IgG (#3900S, CST), anti–MUC1-C (#16564S, CST), anti-STAT1 (#ab109320, Abcam), anti-PBRM1 (A301–591 A, Bethyl Laboratories), anti-H3K27ac (ab4729, RRID:AB_2118291, Abcam), anti-H3K4me1 (ab8895, RRID:AB_306847, Abcam), anti-H3K4me3 (ab8580; RRID:AB_306649, Abcam), anti-JUN (#ab32137, Abcam) and anti-FOS (#2250S, CST) as described.^34,77^

### ATAC-seq analyses

Libraries were generated from three independent replicates each of H1975 and H1975-RT cells, and two replicates each of H1975-RT/CsgRNA and H1975-RT/MUC1sgRNA cells. Library preparation and quality control were performed as described.^29^ The quality of the libraries was assessed using a Tapestation prior to sequencing. Sequencing reads from each replicate were aligned to the human genome (GRCh38) using bwa-mem to generate sorted and deduplicated BAM files. Accessible chromatin regions were identified for each replicate using MACS3 in paired-end mode with an FDR threshold of 0.01.

### Anti-MUC1-C (M1C) ADC sensitivity assays

Cell treatment with M1C ADC and assessment of viability were performed as described^43,60^

### Mouse tumor model studies

H1975-RT, MGH170, and H1975-OR tumor xenografts were established and treated with GO-203 and/or oral osimertinib (MedChemExpress, #HY-15772A) administration at a dose of 10 mg/kg/day.^39^ Established MGH170 PDX tumors were treated with PBS or GO-203 at a dose of 12 μg/gm body weight in the absence and presence of oral osimertinib (OSI) (MedChemExpress, #HY-15772A)^39^ and savolitinib (SAV) administration each at doses of 10 mg/kg/day. MGH170 PDX tumors were treated once weekly x 3 with (1) vehicle control and (2) 7.5 mg/kg M1C ADC. These studies were conducted in accordance with approval by the Dana-Farber Cancer Institute Animal Care and Use Committee (IACUC) under protocol #03–029. Tumor measurements and body weights were recorded twice per week. Protocol-approved experimental and humane endpoints were followed using criteria of tumor size reaching 2 cm in any dimension and 15% weight loss from the last maximum weight measurement. According to the approved protocol guidelines, mice were closely monitored by the DFCI Animal Research Facility (ARF) staff and euthanized before tumors reached 2 cm in diameter.

### Analysis of patient-derived tumor tissues

We retrospectively examined EGFR-mutated NSCLCs from patients treated with osimertinib in the Department of Thoracic Oncology, National Hospital Organization Kyushu Cancer Center, from 2018 to 2022 under IRB No. 2019-82 protocol approval. Immunostaining of tumor cells for MUC1-C (#16564, 1:1000 dilution; CST, heat-induced epitope retrieval, pH 6.0) and STAT1 (#9172, 1:1000 dilution; CST) was evaluated with the Allred scoring system as described.^39,78^

### Statistical analysis

Experiments were performed at least three times. The unpaired two-tailed Student’s t-test was used to assess differences between the mean±SD of two groups. *P*-values were considered significant at *p* < 0.05. GraphPad Prism9 was used for all statistical analyses. Asterisks represent *P ≤ 0.05, **P ≤ 0.01, ***P ≤ 0.001, ****P ≤ 0.0001 with CI = 95%.

## Supplementary information

Supplementary Materials

Vivo source data

## Data Availability

The accession numbers for the RNA-seq and ATAC-seq data are GEO Submissions GSE263757, GSE270995, GSE270997, and GSE294981.
