# Supplementary material for: MUCIN 1 confers inflammatory memory of tyrosine kinase inhibitor resistance in non-small cell lung cancer
Source: Signal Transduct Target Ther. 2025 Nov 28;10:389. doi: 10.1038/s41392-025-02482-7 (PMC12660833; doi:10.1038/s41392-025-02482-7)
Supplement: Supplementary file 1 — Supplementary Materials [file 41392_2025_2482_MOESM1_ESM.docx]

Supplementary Materials for

**tyrosine kinase inhibitor resistance in non-small cell lung cancer**

Shinkichi Takamori^1,#^, Naoki Haratake^1,#^, Atrayee Bhattacharya^1,#,*^, Chie Kikutake^2^, Hiroki Ozawa^1^, Keisuke Shigeta^1^, Ayako Nakashoji^1^, Hideko Isozaki^3^, Mototsugu Shimokawa^4^, Mikita Suyama^2^, Asato Hashinokuchi^5^, Kazuki Takada^6^, Gouji Toyokawa^6^, Yuichi Yamada^7^, Tomoyoshi Takenaka^5^, Kenichi Taguchi^7^, Masafumi Yamaguchi^8^, Tomoharu Yoshizumi^5^,

Aaron N. Hata^3^, and Donald Kufe^1,*^

***Corresponding authors:** [Donald_Kufe@dfci.harvard.edu](mailto:Donald_Kufe@dfci.harvard.edu), [Atrayee_Bhattacharya@dfci.harvard.edu](mailto:Atrayee_Bhattacharya@dfci.harvard.edu), Dana-Farber Cancer Institute, 450 Brookline Avenue, D830, Boston, MA 02215

**This PDF file includes:**

Materials and Methods

Supplementary Text

Figures. S1 to S7

Tables S1 to S2

**Supplementary Figure Supplementary Figure 1. MUC1-C regulates gene signatures in H1975-OR vs H1975 cells. a.** RNA-seq was performed on biologic triplicates of H1975-OR and H1975 cells. Analysis of the H1975-OR vs H1975 transcriptomes using the indicated HALLMARK gene signatures. **b.** GSEA of H1975-OR vs H1975 cell transcriptomes using the HALLMARK INFLAMMATORY RESPONSE gene signature. **c.** Lysates from H1975-OR/tet-MUC1shRNA cells treated with vehicle or DOX for 7 days were immunoblotted with antibodies against the indicated proteins. **d.** Analysis of the datasets from H1975-OR/tet-MUC1shRNA cells treated with DOX vs vehicle using the indicated HALLMARK gene signatures. **e.** H1975-OR cells grown in the absence of osimertinib for 2 to 14 weeks were treated with the indicated concentrations of osimertinib for 3 days and analyzed for cell viability by Alamar Blue staining. The results (mean±SD of 4 determinations) are expressed as relative cell number (% control) compared to that for H1975-OR cells.

**f.** Lysates from H1975-RT/CsgRNA, H1975-RT/MUC1sgRNA and H1975-RT/MUC1sgRNA#2 cells were immunoblotted with antibodies against the indicated proteins. **g.** H1975-RT/CsgRNA, H1975-RT/MUC1sgRNA and H1975-RT/MUC1sgRNA#2 cells treated with the indicated concentrations of osimertinib for 3 days were analyzed for cell viability by Alamar Blue staining. The results (mean±SD of 4 determinations) are expressed as relative cell number (% control) compared to that for H1975-RT/CsgRNA cells. **h.** Purified H1975 cell chromatin left untreated and treated with 20 μM dithiothreitol (DTT) was immunoblotted with antibodies against the indicated proteins.

**Supplementary Figure 2. MUC1-C regulates osimertinib resistance in H1975 and PC9 cells. a.** Analysis of the H1975-RT vs H1975-OR cell transcriptomes using the indicated HALLMARK gene signatures. **b.** H1975-RT cells grown in the presence of 1 μM osimertinib for 1-3 weeks were treated with the indicated concentrations of osimertinib for 3 days and analyzed for cell viability by Alamar Blue staining. The results (mean±SD of 4 determinations) are expressed as relative cell number (% control) compared to that for H1975-RT-OR cells. **c.** H1975-RT/CsgRNA and H1975-RT/MUC1sgRNA cells grown in the presence of 1 μM osimertinib for 2 weeks were treated with the indicated concentrations of osimertinib for 3 days and analyzed for cell viability by Alamar Blue staining. The results (mean±SD of 4 determinations) are expressed as relative cell number (% control) compared to that for H1975/CsgRNA cells. **d.** PC9/vector (left) and PC9/MUC1-C (right) cells were treated with 1 μM osimertinib for 2 weeks. The cells were then treated with the indicated concentrations of osimertinib for 3 days and analyzed for cell viability by Alamar Blue staining. The results (mean±SD of 4 determinations) are expressed as relative cell number (% control) compared to that for PC9/vector cells. **e.** Chromatin from PC9/vector and PC9/MUC1-C cells treated with vehicle or 0.5 μM osimertinib was immunoblotted with antibodies against the indicated proteins. **f.** Summary of (i) H1975, H1975-OR, H1975-RT and H1975-RT-OR cells, and (ii) PC9, PC9/MUC1-C, PC9/MUC1-C-OR with their relative resistance to osimertinib, relative expression of MUC1-C in chromatin and relative expression of STAT1 and ISGs in total lysates as compared to parental cells.

**Supplementary Figure 3. Effects of targeting the MUC1-C/STAT1 auto-regulatory pathway on the memory response to osimertinib treatment. a.** PC9/vector and PC9/MUC1-C cells treated with 0.5 μM osimertinib for 2 days were analyzed for MUC1-C and STAT1 mRNA levels by qRT-PCR. The results (mean±SD of four determinations) are expressed as relative levels compared to that obtained for vector control cells (assigned a value of 1). **b.** H1975-RT/tet-MUC1shRNA cells treated with vehicle or DOX for 7 days and then with 1 μM osimertinib for 2 days were analyzed for MUC1-C and STAT1 mRNA levels by qRT-PCR. The results (mean±SD of four determinations) are expressed as relative levels compared to that obtained for control cells (assigned a value of 1). **c.** Lysates from H1975-RT/CshRNA and H1975-RT/STAT1shRNA were immunoblotted with antibodies against the indicated proteins. **d.** H1975-RT/CshRNA and H1975-RT/STAT1shRNA cells exposed to 1 μM osimertinib for 2 weeks were analyzed for osimertinib sensitivity by Alamar Blue staining. The results (mean±SD of 4 determinations) are expressed as relative cell number (% control) compared to that for H1975-RT/CshRNA cells. **e.** H1975-OR (OSI IC50=5.6 μM) cells were grown in the absence of osimertinib for 12 weeks (drug holiday) to establish H1975-RT cells (OSI IC50=1.4 μM). Treatment of H1975-RT cells with osimertinib for 2 days was associated with upregulation of MUC1-C, STAT1 and ISG expression (left). These results in osimertinib-treated H1975-RT cells support an inflammatory memory response that is activated by the auto-inductive MUC1-C/STAT1 pathway which drives ISG expression and promotes osimertinib resistance (right). Image generated using BioRender software.

**Supplementary Figure 4. Effects of targeting JUN/AP-1 on the response of H1975-RT cells to osimertinib treatment. a.** Soluble chromatin from H1975 cells treated with 1 μM osimertinib for 3 days was precipitated with antibodies against the indicated proteins or a control IgG. The DNA samples were amplified by qPCR for pELS-2. The results (mean±SD of 3 determinations) are expressed as percent input. **b.** H1975-RT/tet-AFOS cells treated with vehicle or DOX for 7 days and then 1 μM osimertinib for 2 days were analyzed for the indicated mRNA levels by qRT-PCR (left)**.** The results (mean±SD of 4 determinations) are expressed as relative levels compared to that obtained for vehicle-treated cells (assigned a value of 1). Lysates were immunoblotted with antibodies against the indicated proteins (right)**. c.** H1975-RT/tet-AFOS cells treated with vehicle or DOX for 7 days and then 1 μM osimertinib for 2 days were analyzed for the indicated mRNA levels by qRT-PCR (left)**.** The results (mean±SD of 4 determinations) are expressed as relative levels compared to that obtained for vehicle-treated cells (assigned a value of 1). Lysates were immunoblotted with antibodies against the indicated proteins (right)**. d.** H1975-RT/tet-AFOS cells treated with vehicle or DOX for 7 days and exposed to osimertinib for 2 weeks were analyzed for osimertinib sensitivity by Alamar Blue staining. The results (mean±SD of 4 determinations) are expressed as relative cell number (% control) compared to that for vehicle-treated H1975-RT/tet-AFOS cells. **e.** Lysates from H1975-RT/tet-MUC1shRNA cells treated with vehicle or DOX for 7 days and then 1 μM osimertinib for 2 days were immunoblotted with antibodies against the indicated proteins. **f.** Lysates from H1975-RT cells treated with vehicle or GO-203 for 2 days and then 1 μM osimertinib for 2 days were immunoblotted with antibodies against the indicated proteins. **g.** PC9/vector and PC9/MUC1-C cells treated with 0.5 μM osimertinib for 2 days were analyzed for the indicated mRNA mRNA levels by qRT-PCR. The results (mean±SD of 4 determinations) are expressed as relative levels compared to that obtained for untreated H1975-RT cells (assigned a value of 1). **h.** Summary of relative changes in JUN and FOS expression in (i) H1975 cells without and with OSI treatment for 2 days, (ii) H1975-RT cells without and with OSI treatment for 2 days, and (iii) H1975-RT with MUC1-C targeting (upper). Proposed model for increased occupancy of the *MUC1* pELS-2 by MUC1-C/JUN in H1975-RT cells and by MUC1-C/JUN/FOS in the memory response to OSI re-exposure (lower). Induction of MUC1-C expression promotes STAT1-mediated upregulation of inflammatory memory ISGs. Image generated using BioRender software.

**Supplementary Figure 5. Effects of targeting PBRM1 on the response of H1975-RT cells to osimertinib treatment. a.** ATAC-seq was performed in (i) triplicates on H1975 and H1975-RT cells, and (ii) duplicates on H1975-RT/CsgRNA and H1975-RT/MUC1sgRNA cells. Representative genome browser snapshots are shown for the indicated MUC1-C-induced ISGs. Highlighted are *IRF1* and *ISG15* promoter regions activated by STAT1 signaling. Distinct peaks of chromatin accessibility were not detectable in the *MX1* and *OAS1* genes. **b.** GSEA of H1975-OR vs H1975 cell RNA-seq data using the GOCC SWI SNF Complex gene signature. **c.** GSEA of RNA-seq data from H1975-OR/tet-MUC1shRNA cells treated with vehicle or DOX for 7 days using the GOCC SWI SNF Complex gene signature. **d.** Lysates from H1975-RT cells treated with vehicle or 1 μM osimertinib for 2 days were immunoblotted with antibodies against the indicated proteins. **e.** H1975 and H1975-RT cells treated with 1 μM osimertinib for 2 days were analyzed for ARID1A mRNA levels by qRT-PCR. The results (mean±SD of 4 determinations) are expressed as relative levels compared to that obtained for untreated cells (assigned a value of 1). **f.** H1975-RT/tet-MUC1shRNA cells treated with vehicle or DOX for 7 days and then exposed to 1 μM osimertinib for 2 days were analyzed for BRG1 and PBRM1 mRNA levels by qRT-PCR (left). The results (mean±SD of four determinations) are expressed as relative levels compared to that obtained for vehicle-treated H1975 cells (assigned a value of 1). Lysates were immunoblotted with antibodies against the indicated proteins (right). **g.** H1975-RT cells treated with GO-203 for 2 days and then 1 μM osimertinib for 2 days were analyzed for BRG1 and PBRM1 mRNA levels by qRT-PCR (left). The results (mean±SD of four determinations) are expressed as relative levels compared to that obtained for untreated H1975-RT cells (assigned a value of 1). Lysates were immunoblotted with antibodies against the indicated proteins (right). **h.** PC9/vector and PC9/MUC1-C cells treated with 0.5 μM osimertinib for 2 days were analyzed for BRG1 and PBRM1 mRNA levels by qRT-PCR. The results (mean±SD of determinations) are expressed as relative levels compared to that obtained for untreated H1975-RT cells (assigned a value of 1). **i.** H1975-RT/CshRNA and H1975-RT/PBRM1shRNA cells treated with 1 μM osimertinib were analyzed for clonogenic survival. Shown are representative photomicrographs of stained colonies. The results (mean±SD of three determinations) are expressed as relative colony formation compared to that for untreated cells (assigned a value of 1). **j.** Treatment of H1975-RT vs H1975 cells with OSI induces the memory response as exemplified by activation of the *MUC1* pELS-2 region. MUC1-C, AP-1 and PBAF/PBRM1 occupancy of pELS-2 is induced in association with MUC1-C-dependent increases in chromatin accessibility and deposition of H3K27ac and H3K4me1. In turn, MUC1-C expression is upregulated in an auto-inductive pathway that drives STAT1 and ISGs expression. Image generated using BioRender software.

**Supplementary Fig. 6. MUC1-C dependency of patient-derived MGH170 and MGH121 NSCLC cells with acquired osimertinib resistance. a.** MGH170/MUC1shRNA cells treated with vehicle or DOX for 7 days and then with 1 μM osimertinib for 3 days were analyzed for the indicated mRNA levels by qRT-PCR. The results (mean±SD of four determinations) are expressed as relative levels compared to that obtained for untreated cells (assigned a value of 1). **b.** MGH170 cells treated with the indicated osimertinib concentrations alone and in combination with 0.5 μM capmatinib (CAP) or 0.5 μM savolitinib (SAV) for 3 days were analyzed by Alamar Blue staining. The results (mean±SD of 4 determinations) are expressed as relative cell number (% control) compared to that for control cells. **c.** MGH170 cells treated with 0.5 μM osimertinib and/or 0.5 μM capmatinib for 2 days were analyzed for the indicated mRNA levels by qRT-PCR. The results (mean±SD of 4 determinations) are expressed as relative levels compared to that obtained for untreated cells (assigned a value of 1). **d.** MGH170/tet-MUC1shRNA cells treated with vehicle or DOX for 7 days and then with 0.5 μM osimertinib and/or 0.5 μM capmatinib for 2 days were analyzed for the indicated mRNA levels by qRT-PCR. The results (mean±SD of 4 determinations) are expressed as relative levels compared to that obtained for untreated cells (assigned a value of 1). **e.** MGH170 cells treated with vehicle or 1 μM GO-203 were analyzed for indicated mRNA levels by qRT-PCR. The results (mean±SD of four determinations) are expressed as relative levels compared to that obtained for untreated cells (assigned a value of 1). **f.** MGH121 cells treated with vehicle or 5 μM GO-203 for 2 days and then 1 μM TQB3804 for 2 days were analyzed for the indicated mRNA levels by qRT-PCR. The results (mean±SD of 4 determinations) are expressed as relative levels compared to that obtained for untreated cells (assigned a value of 1). **g.** MGH121 cells were treated with the indicated concentrations of osimertinib alone, TQB3804 alone and TQB3804+GO-203 for 2 days and analyzed for cell viability by Alamar Blue staining. The results (mean±SD of 4 determinations) are expressed as relative cell number (% control) compared to that for control MGH121 cells and as IC50 μM values.

**Supplementary Fig. 7. Expression of MUC1-C in EGFR mutant NSCLCs associates with response to osimertinib treatment a.** Body weights expressed as the mean±SD for 6 mice bearing MGH170 PDX tumor xenografts treated with control vehicle or M1C ADC. **b.** Representative MUC1-C IHC images of EGFR mutant NSCLC tumors prior to osimertinib treatment (upper, low expression; lower, high expression) **c and d.** Clinical characteristics of patients with EGFR mutant NSCLCs treated with osimertinib according to MUC1-C low and high levels **(c)** and univariate analysis of factors associated with PFS and OS **(d).** **e.** Multivariate analysis of factors associated with PFS and OS. **f.** Representative images of STAT1 IHC staining of EGFR mutant NSCLC tumors prior to osimertinib treatment (upper, low expression; lower, high expression).

**Supplementary Tables**

**Supplementary Table 1. Primers used for qRT-PCR analysis.**

| **MUC1-C** | **FWD** | TACCGATCGTAGCCCCTATG |
| --- | --- | --- |
|  | **REV** | CTCACCAGCCCAAACAGG |
| **STAT1** | **FWD** | GGAACTTGATGGCCCTAAAGGA |
|  | **REV** | ACAGAGCCCACTATCCGAGACA |
| **OAS1** | **FWD** | TGAGGTCCAGGCTCCACGCT |
|  | **REV** | GCAGGTCGGTGCACTCCTCG |
| **MX1** | **FWD** | CTTTCCAGTCCAGCTCGGCA |
|  | **REV** | AGCTGCTGGCCGTACGTCTG |
| **ISG15** | **FWD** | CGCAGATCACCCAGAAGATCG |
|  | **REV** | TTCGTCGCATTTGTCCACCA |
| **PBRM1** | **FWD** | AAGAAGAAAGAGCTTGCCAG |
|  | **REV** | TCTCGAGCTTCAAGAACAAC |
| **BRG1** | **FWD** | CCAAGACCCTGATGAACACC |
|  | **REV** | GGCAGAACAGCAGCACTTT |
| **ARID1A** | **FWD** | ACCTCTATCGCCTCTATGTGTCTGT |
|  | **REV** | CTGGCAGCACTGCTTGATGT |
| **β-actin** | **FWD** | GATGAGATTGGCATGGCTTT |
|  | **REV** | CACCTTCACCGTTCCAGTTT |

**Supplementary Table S2. Primers used for ChIP-qPCR.**

| **MUC1-C pELS-1** | **FWD** | GCTGGAGAACAAACGGGTAG |
| --- | --- | --- |
|  | **REV** | GAGCAGGTGACAGGTGACAA |
| **MUC1-C pELS-2** | **FWD** | CGGAGAAAACACGAGTAGCTAG |
|  | **REV** | TTTAAGTACTCAGACTGCGCG |
